# Insights into the liver-eyes connections, from epidemiological, mechanical studies to clinical translation

**DOI:** 10.1186/s12967-023-04543-3

**Published:** 2023-10-10

**Authors:** Junhao Wu, Caihan Duan, Yuanfan Yang, Zhe Wang, Chen Tan, Chaoqun Han, Xiaohua Hou

**Affiliations:** 1 Division of Gastroenterology, Union Hospital, Tongji Medical College, Huazhong University of Science and Technology, 1277 Jiefang Avenue, Wuhan, 430022 Hubei China; 2 State Key Laboratory of Ophthalmology, Zhongshan Ophthalmic Centre, Sun Yat-Sen University, Guangdong Provincial Key Laboratory of Ophthalmology and Visual Science, Guangzhou, China

**Keywords:** Liver-eye axis, Interorgan  communication, Pathogenesis and homeostasis, Therapeutic strategies

## Abstract

Maintenance of internal homeostasis is a sophisticated process, during which almost all organs get involved. Liver plays a central role in metabolism and involves in endocrine, immunity, detoxification and storage, and therefore it communicates with distant organs through such mechanisms to regulate pathophysiological processes. Dysfunctional liver is often accompanied by pathological phenotypes of distant organs, including the eyes. Many reviews have focused on crosstalk between the liver and gut, the liver and brain, the liver and heart, the liver and kidney, but with no attention paid to the liver and eyes. In this review, we summarized intimate connections between the liver and the eyes from three aspects. Epidemiologically, we suggest liver-related, potential, protective and risk factors for typical eye disease as well as eye indicators connected with liver status. For molecular mechanism aspect, we elaborate their inter-organ crosstalk from metabolism (glucose, lipid, proteins, vitamin, and mineral), detoxification (ammonia and bilirubin), and immunity (complement and inflammation regulation) aspect. In clinical application part, we emphasize the latest advances in utilizing the liver-eye axis in disease diagnosis and therapy, involving artificial intelligence-deep learning-based novel diagnostic tools for detecting liver disease and adeno-associated viral vector-based gene therapy method for curing blinding eye disease. We aim to focus on and provide novel insights into liver and eyes communications and help resolve existed clinically significant issues.

## Introduction

Over the last decades, more and more researches have raised human understanding of interorgan connections to an unprecedented level, particularly in the fields of epidemiology, molecular biology, diagnostics, and therapeutics.

As the largest solid organ, the liver performs a number of essential bioactivities related to metabolism, immunity, endocrine, storage, and detoxification, implying its central role in systemic regulation. Meanwhile, this could become a double-edged sword, as liver dysfunction would disrupt homeostasis and significantly influence extrahepatic tissues, including the eyes [1]. From an ocular perspective, its unique anatomical structures have been utilized as a window to directly witness disease-related neurovascular changes, and the latest study further expands this application to detecting asymptomatic liver disease like fatty liver disease, viral hepatitis, and slight cirrhosis even at the initial stages [2]. Such timely identification plus interventions can significantly improve the disease prognosis [3]. However, currently published reviews about the liver or eye rarely focus on interorgan connections, which is far behind the increasing number of basic medical studies and clinical practices.

Therefore, finding out the underlying molecular pathways between the liver and the eyes, as well as their change rules, embodiment in the epidemiological/clinical relationship could help to deepen our insights into the occurrence/progression of liver-eye diseases, and subsequently into their diagnosis/treatment.

## Epidemiological evidence and public health significance

An increasing number of epidemiological works indicate that eye disease has liver-related protective factors or risk factors and *vice versa* [4–7]. A timely identification of such factors would help health care workers better evaluate patients’ status and better identify high-risk groups of liver/eye disease patients that require a transfer to receive complete ophthalmic/hepatic examination, which is certainly beneficial to individual outcomes [8]. Besides, an early alert as well as intervention could significantly reduce disease burden for society [9], particularly in an era with an aging and growing population when blinding ophthalmic conditions and all types of liver diseases are becoming prevalent [9, 10].

### Ophthalmopathy-related hepatic factors

**Fig. 1:**
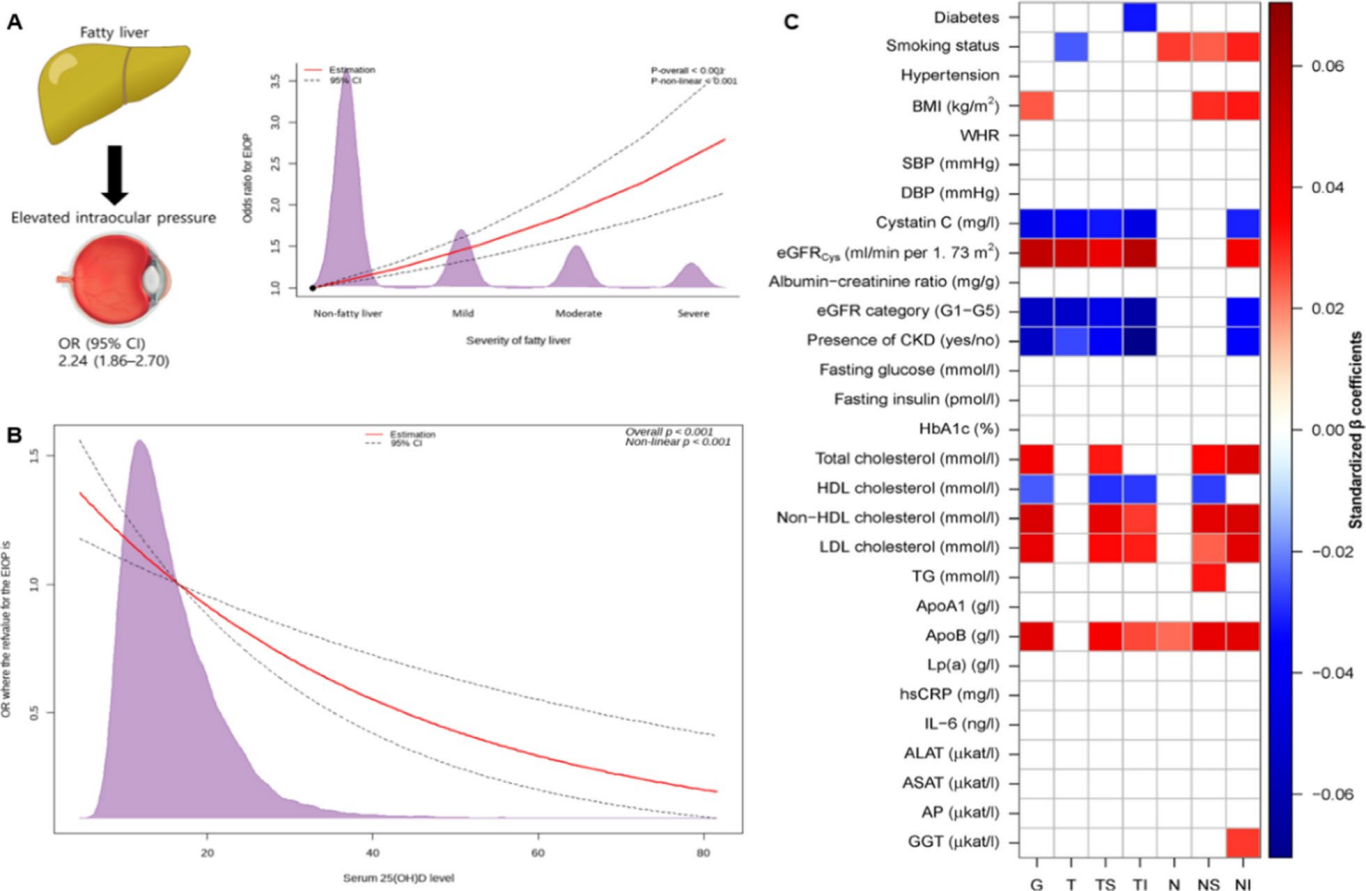
Gluacoma-related hepatic factors. **A** The restricted cubic spline curve shows a linear dose-response relationship between ORs for EIOP and the severity of fatty liver. The red line indicates the estimated OR, and the dotted lines indicate the 95% CI. The purple areas indicate population density. *ORs* odds ratios, *EIOP* elevated intraocular pressure, *CI* confidence interval. p < 0.05 implies statistically significant. This graph is cited from the “Graphic abstract” in [11] without any adaptation, Lee, Jun-Hyuk et al., Fatty Liver Is an Independent Risk Factor for Elevated Intraocular Pressure. Nutrients. 2022; 14(21):4455, with the permission from the Creative Commons Attribution (CC BY) license (https://creativecommons.org/licenses/by/4.0/). **B** This cubic spline curve illustrates an inverse dose-response relationship between the plasma 25(OH)D levels and ORs of EIOP. *25(OH)D* 25-hydroxyvitamin D3, *ORs*, odds ratios. This illustration is cited from Fig. 3 in [12] without any adaptation, Lee, Jun-Hyuk et al., Inverse Relationship between Serum 25-Hydroxyvitamin D and Elevated Intraocular Pressure. Nutrients. 20–23; 15(2):423, with permission from the Creative Commons Attribution (CC BY) license (https://creativecommons.org/licenses/by/4.0/). **C** A standardized β coefficients heatmap for multiple biomarkers with global and sectoral cpRNFLT. Multivariable linear regression analysis is carried out for each biomarker (independent variable) with corresponding global and sectoral cpRNFLT (dependent variable) with age, sex, and scanning circle radius adjustment. White (empty) squares are depicted for sectors that do not come to a valid corrected p value by the false-positive discovery rate method, while positive associations (red colored), and negative associations (blue colored) are shaded based on respective standardized β coefficients for those significant sectors. *GGT*, γ-glutamyl transferase, *WHR*, waist-to-hip ratio, *HDL*, high-density lipoprotein, *ApoB*, apolipoprotein B, Optic nerve head sectors: *LDL*, low-density lipoprotein, *N*, nasal sector, *NI*, infero-nasal sector, *NS*, supero-nasal sector, *TI*, infero-temporal sector, *T*, temporal sector, *TS*, supero-temporal sector, *G*, global sector. This heatmap is cited from Fig. 1 in [17] without any adaptation, Rauscher, F.G., Wang, M., Francke, M. et al. Renal function and lipid metabolism are major predictors of circumpapillary retinal nerve fiber layer thickness—the LIFE-Adult Study. BMC Med 19, 202 (2021), with permission under the Creative Commons International licence 4.0. (http://creativecommons.org/licenses/by/4.0/)

Glaucoma is the main reason for irreversible blindness worldwide and is estimated to afflict more than 76 million people [11]. Elevated intraocular pressure (EIOP) is the only modifiable risk factor identified to date during the whole course [12]. Retrospective studies have indicated that mean IOP levels in Asian adults are positively and linearly increased with the nonalcoholic-fatty liver disease (NAFLD) grades [5], and therefore liver steatosis patients have elevated odds ratios (ORs) for high IOP (≥ 22 mmHg), which shows a linear dose-response relationship with the severity of fatty liver, including in patients with alcoholic liver disease [13, 14]. The cubic spline curve from another cross-sectional study indicates that there is an inverse dose-dependent relationship between ORs for EIOP and serum 25-hydroxyvitamin D3(25(OH)D) content, especially in subgroups < 20 ng/ml [15]. Given that more than 37% of the global population might suffer from NAFLD [16], up to 24% and 40% of the people, respectively, in the US and Europe are estimated to suffer from vitamin D deficiency [17], and nearly 9.5% of untreated EIOP patients would deteriorate into primary glaucoma over the next 5-year follow-up [18], physicians may well raise their alertness. In addition to EIOP, circumpapillary retinal nerve fiber layer thickness (cpRNFLT) thinning has been identified as one of the well-performed initial signs of glaucoma [19, 20]. The LIFE-Adult study also suggests categorizing adverse lipid profiles (e.g., high apolipoprotein B (ApoB), high non-HDL cholesterol, high total cholesterol, high low-density lipoprotein (LDL) cholesterol, low high-density lipoprotein (HDL) cholesterol) as independent covariates of thicker cpRNFLT, thus helping conceal nerve fiber decay in glaucoma, of which per 1 mmol/l increase in non-HDL cholesterol brings about 0.5 μm elevation in cpRNFLT [20]. Quantifying the impacts of adverse liver-lipid metabolism profiles matters to refining cpRNFLT-based early diagnosis of glaucoma. (Fig. 1).

**Fig. 2:**
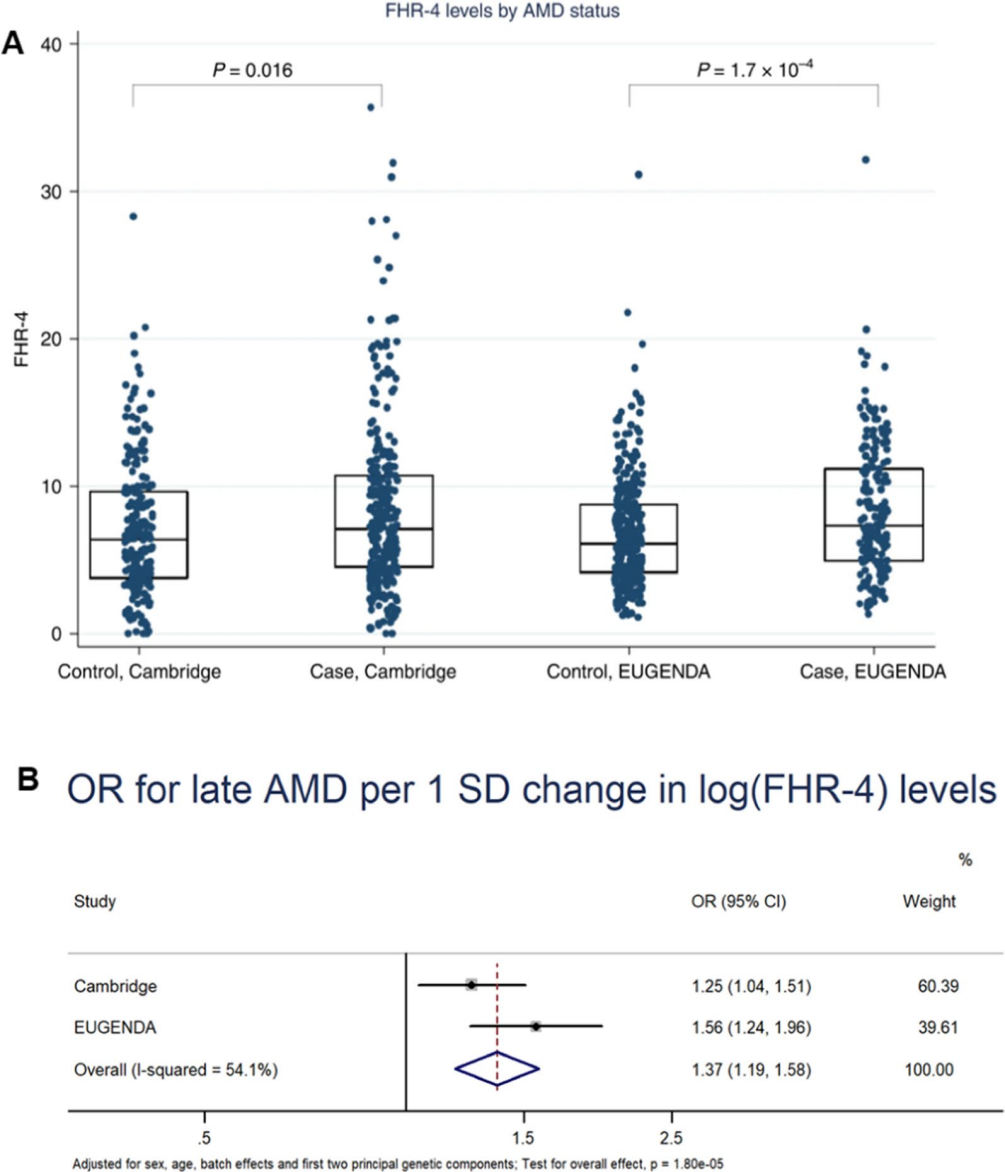
AMD-associated liver factor FHR-4. **A** Box plots show serum FHR-4 levels from two independent cohorts: Cambridge (214 controls and 304 advanced AMD cases) and EUGENDA (308 controls with 180 advanced cases). The geometric mean FHR-4 levels are: in Cambridge, 5.5 µg ml/l in controls and 6.6 µg ml/l in AMD cases; in EUGENDA, 6.0 µg ml/l in controls and 7.2 µg ml/l in cases. AMD patients have statistically increased FHR-4 levels compared to controls, and these differences still remain significant after sex, age, batch effects, and the first two genetic principal components adjustment (p = 0.018 and 8.4 × 10^−5^, for Cambridge and EUGENDA, respectively; Wald test). *AMD* age-related macular degeneration, *FHR-4* Factor H-related protein 4. **B** A two-stage, fixed-effects meta-analysis of individual participants’ data from the Cambridge and EUGENDA study shows a significant association between FHR-4 levels and late AMD. Panel A shows forest plots of ORs with 95% CI of late AMD per SD change in natural logarithmically transformed FHR-4 levels using logistic regression models after sex, age, batch effects, and the first two genetic principal components adjustment. The overall OR estimate is obtained from a two-stage, fixed-effects, meta-analysis of the two study-specific estimates. I^2^ statistics is used to assess heterogeneity across studies. *OR* odds ratios, *CI* confidence intervals, *SD* standard deviation. **A** and **B** are respectively adapted from Fig. 1 and Fig. S1 in [16], Cipriani, V., Lorés-Motta, L., He, F. et al. Increased circulating levels of Factor H-Related Protein 4 are strongly associated with age-related macular degeneration. Nat Commun 11, 778 (2020), with authorization from the Creative Commons International licence 4.0 (http://creativecommons.org/licenses/by/4.0/)

Age-related macular degeneration (AMD) is another leading cause of blindness in elderly people and shows a prominent genetic basis [21]. AMD patient cohort studies have demonstrated the positive correlation between high levels of serum Complement Factor H-related protein 4 (FHR-4) and the risk of AMD [4]. Such an increase in FHR-4 is related with locus variants, such as rs10922109 of Factor H (CFH), that can upregulate its liver expression level [22]. The Genome-Wide Association Study (GWAS) further indicates that hepatic lipase (LIPC) locus variants, especially rs10468017 of the promoter, are related to late AMD [23] (Fig. 2).

Diabetic retinopathy (DR) is a very common and blinding complication of diabetes mellitus affecting more than 100 million individuals worldwide, which is accompanied by dyslipidemia normally [24, 25]. A cross-sectional research has validated that, for patients with type II diabetic mellitus (T2DM), those having a higher cholinesterase (CHE) level (> 10,500 U/L) are at a lower OR of DR at 0.498, while those with a lower total proteins level (< 60 g/L) have a higher OR at 1.624 [26]. Another 10-year prospective cohort study further indicates that plasma ApoA-I level (≥ 7.4 μmol/L) is correlated with a decreased hazard ratio (HR) of DR at 0.86, while serum ApoC-III (≥ 6.3 μmol/L) and ApoE levels (≥ 1.1 μmol/L) are correlated with a higher HR at about 1.2 [24]. Besides, serum lipoprotein(a) (≥ 30.5 mg/dL) is related with an adjusted incidence OR of DR at 3.46, while increased levels of ApoB (≥ 77.5 g/L) and fetuin-A (a major liver-derived glycoprotein) present a positive correlation with DR degree at an adjusted OR = 1.02 [27, 28]. Liver derived peptide adropin shows the opposite trend [1, 29]. NAFLD is related with proliferative/laser-treated DR at an adjusted OR = 1.75 [30]. Given that apolipoproteins could reflect liver functions and are not influenced by dietary status [24], taking fundus examination in T2DM patients who have the above-mentioned changes could help DR monitoring.

Patients with dry eye disease (DED) present with ocular surface inflammation and tear film homeostatic imbalance [31], at a prevalence rate ranging from 5% to 50% within different populations [32]. The Lifelines Cohort study demonstrates that liver cirrhosis and gallstone are independent risk factors of DED, with respective OR at 3.38 and 1.22 [33].

Ocular motor cranial nerve palsies (CNP) paralyze extra-ocular muscles and cause diplopia [34]. Data from National Health Insurance Service-National Sample Cohort (NSC) shows that adults with low HDL cholesterol, elevated triglyceride, and elevated alanine aminotransferase (ALT) contents have a higher hazard ratio (HR) for ocular motor CNP at 1.24, 1.18, and 1.141, respectively [6, 34]. Besides, an increase in the morbidity of ocular motor CNP is observed as the plasma level of liver γ-glutamyl transferase (GGT) increases, with the highest HR at 1.245 [6]. It is worth noting that palsies of the 3rd, 4th, and 6th cranial nerve are rather common in neuro-ophthalmology practice, and GGT is one of the most sensitive indicators for liver function changes [6]. Moreover, it has been found that hepatic cirrhosis patients are prone to smooth pursuit eye movements (SPEM) disruption [35].

Furthermore, NAFLD is identified as a risk factor (OR = 2.378) of vascular lesions, like arteriovenous compression and arterial narrowing in the retina [36]. AFP and CA-125 cut-off values at 957.2 ng/ml and 114.25 U/ml could serve as independent risk factors for predicting ocular metastasis of liver cancer with the separate value of area under the curve (AUC) at 0.739 and 0.810 [37]. However, the two indices may have limited reference value as they are also elevated upon liver carcinogenesis or systemic cancer metastasis [38, 39]. Recently, a Mendelian randomization case-control study reports that genetic variants within the CYP2R1 locus would reduce liver 25-hydroxylase activities and the resultant low blood levels of 25(OH)D are linked with increased non-infectious uveitis/scleritis risk at an OR = 6.42 [40]. Interestingly, excessive blood unbound bilirubin is suggested to be a protective factor in specific conditions, including in neonates to reduce the severity of retinopathy of prematurity (ROP) [41], and in patients with diabetic mellitus or impaired glucose tolerance to protect against DR [42].

### Eye indicators connected with liver status

Similarly, some ocular examination indices can also alert or monitor liver diseases development, which matters to those asymptomatic or rapidly-progressing in the course.

Acute liver failure (ALF) refers to a rare but lethal condition that normally impacts individuals without preexisting liver diseases [8], accompanied by the rapid development of coagulopathy and hepatic encephalopathy secondary to the liver injuries [8]. Complex infectious (i.e., reactivation of chronic hepatitis B virus infection), pharmacological (i.e., acetaminophen), immunological (i.e., autoimmune hepatitis), along with genetic factors (i.e., acute presentations of Wilson disease) could lead to this condition [8]. The prognosis of ALF is correlated with the management of intracranial hypertension [(ICH), referring to constant intracranial pressure > 20 mm Hg] that may well rapidly progress to cerebral herniation and death [8, 43]. Conventional monitoring techniques for ICH, such as CT scan and intracranial catheter insertion, present great false-negative, bleeding and infectious risks [44, 45]. Continuous efforts have been made to develop novel non-invasive techniques for monitoring changes in intracranial pressure and identifying an elevation of it during therapy, timely and accurately [8]. Due to connected subarachnoid space around the optic nerve and the brain, elevated intracranial pressure could be transmitted to the perineural space through cerebrospinal fluid circulation [46], leading to an enlargement in optic nerve sheath diameter (ONSD) [44, 47]. Prior studies of traumatic brain injury-caused intracranial hypertension showed the credibility of measuring ONSD [48]. For its performance in ALF patients, a meta-analysis including 31 studies indicated a higher sensitivity (0.92 versus 0.70; p < 0.01) with an equal specificity in diagnosing elevated intracranial pressure by measuring ONSD using ocular ultrasonography (US) than magnetic resonance imaging (MRI) [49]. Recently, a prospective study performed in adult cohort with ALF reported an increase in median ONSD with hepatic encephalopathy grade [44]. A children ALF cohort also showed a similar trend and further defined thresholds for different stages of hepatic encephalopathy grade [47] (Table 1). These results demonstrated the potential of ONSD in predicting ALF-related prognosis; however, its performance in such small-scale cohorts requires further validation by more large-scale prospective studies [50].

**Table 1:** ONSD cut-off values for identifying high-risk ALF patients

Index	Subjects	Sensitivity (%)	Specificity (%)	Cut-off value (mm)
ONSD	Adult with ALF	100	46.2	> 5.0
		71.4	84.6	> 6.0
	Normal children^a^	–	–	< 4.5
	Children with HE^a^	82	87.5	> 4.6
	Children with grade III/IV HE^a^	82.8	73.3	> 4.9
	Children with poor outcomes^a^	80	80	> 5.1
	Children with emergencies^a^	–	–	> 5.4

*ALF* acute liver failure, *HE* hepatic encephalopathy, *ONSD* the Optic Nerve Sheath Diameter

^a^Children refers to > 4 years old

Infection of hepatitis virus would lead to chronic hepatitis, cirrhosis, and even liver carcinoma, in which the hepatitis C virus (HCV) affects 2.2% of the global population [51], with DED being a common comorbidity. Studies have found that, compared to hepatitis C patients at initial 0–3 stages of cirrhosis, patients at more advanced 4–6 stages tend to have worse ocular surface indices, such as higher Ocular Surface Disease Index (OSDI), decreased Schirmer test I, lower tear-film breakup time, and worse conjunctival/corneal vital dye staining scores [52, 53]. In addition, chorioretinal structures of the eyes could serve as a noninvasive proxy of hepatic microvasculature due to correlations between reduced retinal thickness/macular volumes and increased cirrhosis severity irrelevant to primary etiologies [54]. Particularly, there is an inverse correlation between increased ganglion cell complex (GCC) thickness and Fibrosis-4 scores (a liver fibrosis risk index) when above the minimum cut-off value (score ≥ 2.67) in elderly people (defined as > 65 years old) [55].

NAFLD is estimated to take the place of the hepatitis B and C viruses as the driving cause of hepatocellular carcinoma (HCC) [7]. Data from a case-control work indicates that DR is an independent risk factor for HCC development (OR = 8.654) in patients having NAFLD, and these high-risk groups should therefore have regular HCC screening [7]. For children with NAFLD, liver fibrosis degrees and retinopathy sign severity (pathological grading for retinal arterial tortuosity) display a positive correlation (r = 0.31) [56].

**Fig. 3:**
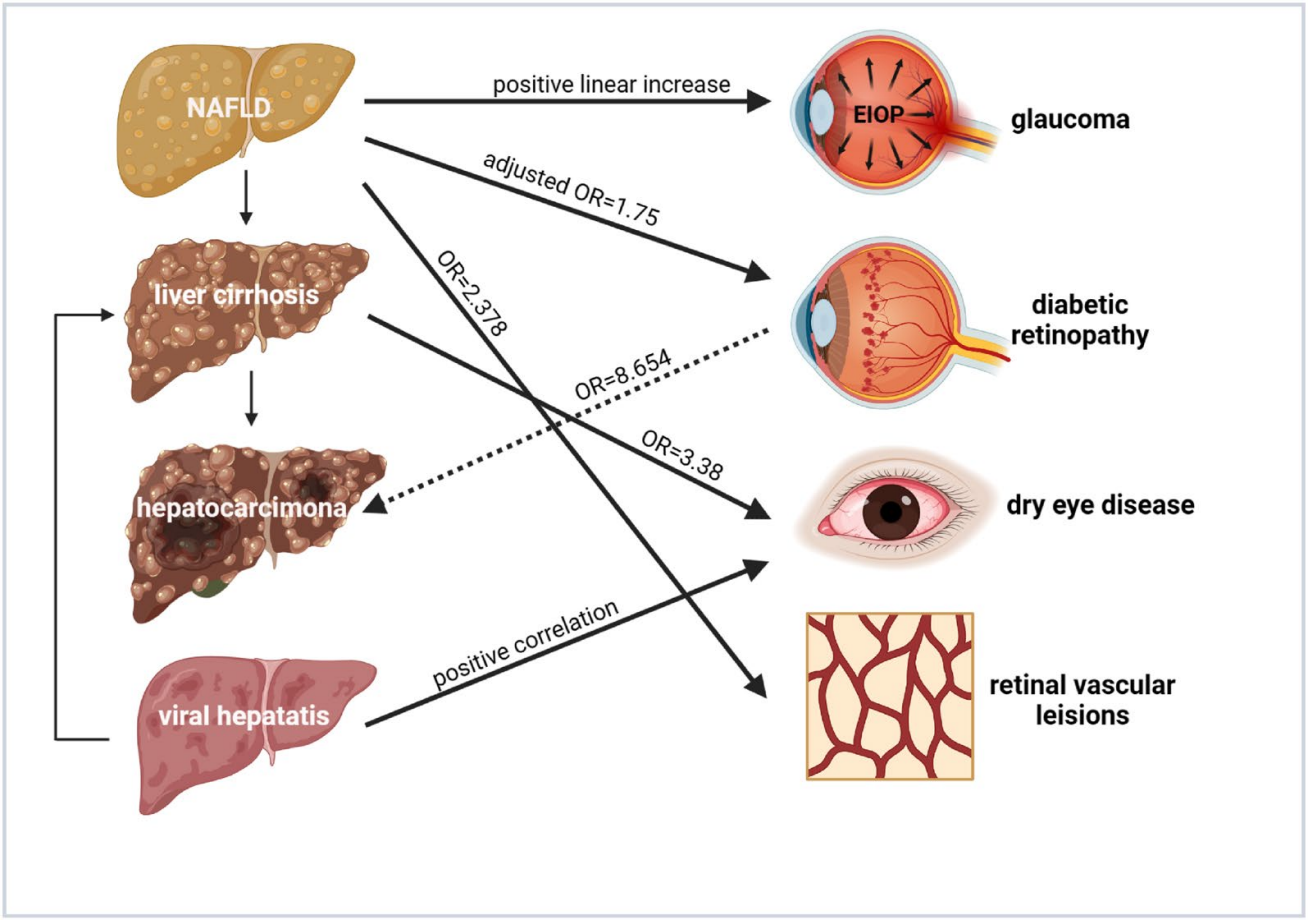
The liver-eye epidemiological correlation network. Common liver diseases listed (left) are linked with their epidemiologically correlated ocular pathologies (right). NAFLD is associated with glaucoma, diabetic retinopathy, and dry eye disease, whereas viral hepatitis and cirrhosis are linked with dry eye disease. In turn, diabetic retinopathy could elevate hepatocarcinoma risk in T2DM patients. *NAFLD* nonalcoholic fatty liver disease, *EIOP* elevated intraocular pressure, *T2DM* type II diabetic mellitus, *OR* odds ratio. Created with Biorender.com

The mutual epidemiological correlations between the liver and eyes were depicted in Fig. 3.

## Molecular pathways and pathophysiological regulation

**Fig. 4:**
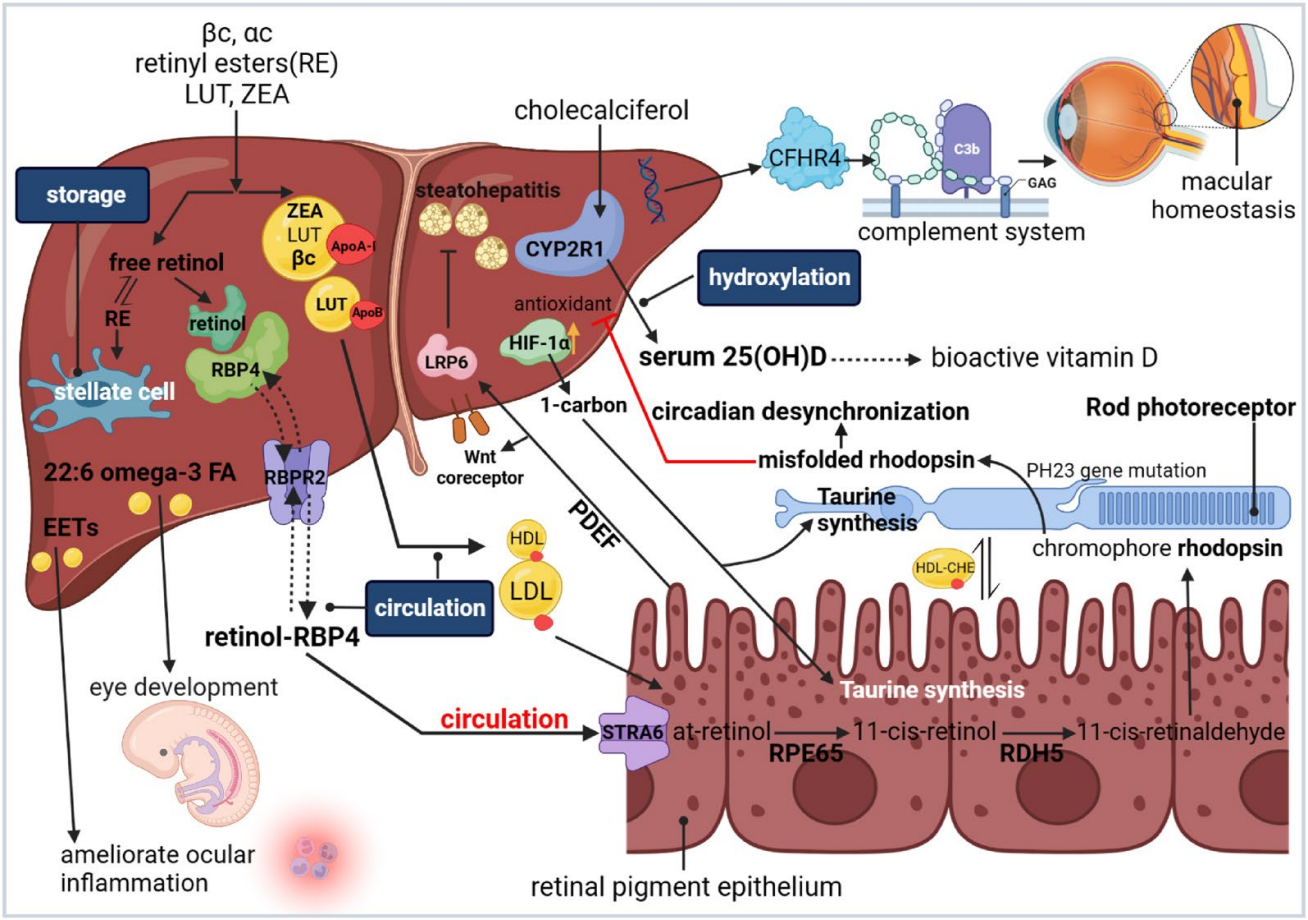
The molecular liver-eye regulatory pathways under physiological settings. (Taken normal liver as an example.). *LUT* lutein, *ZEA* zeaxanthin, *βc* β-carotene, *αc* α-carotene, *CFHR4* Factor H-related protein 4, *25(OH)D*, 25-hydroxyvitamin D3, *FA* fatty acid, *HDL* high density lipoprotein, *LDL* low-density lipoprotein, *EET* epoxyeicosatrienoic acid, *PEDF* epithelium-derived factor, *RE* retinyl esters. Created with Biorender.com

**Fig. 5:**
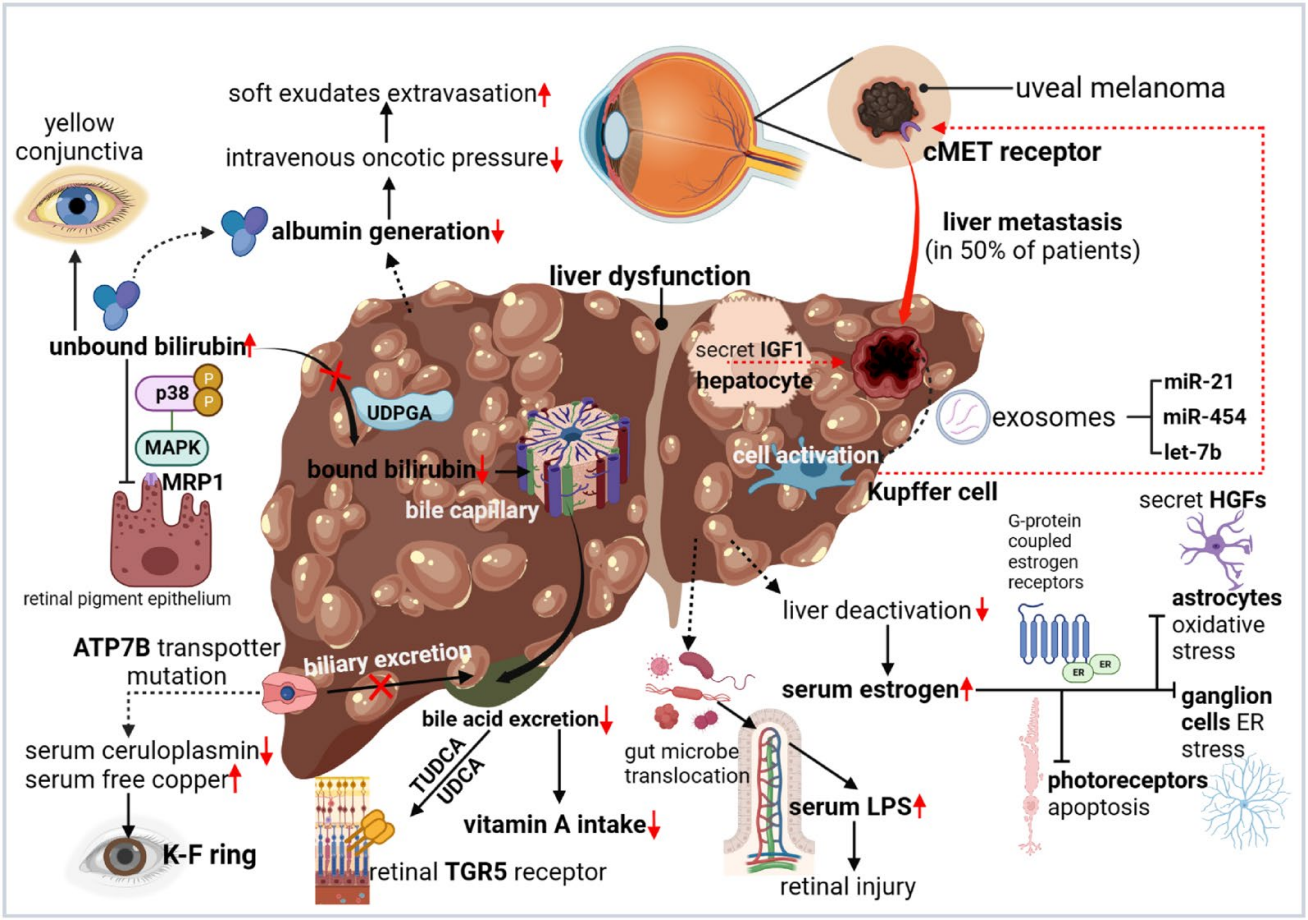
Underlying interorgan molecular pathways changes in pathological conditions. (Taken liver cirrhosis as an example.) *RPE* retinal pigment epithelium, *TUDCA* tauroursodeoxycholic acid, *UDCA* ursodeoxycholic acid, *HGF* hepatocyte growth factor, *IGF* insulin-like growth factor 1, *LPS* lipopolysaccharide, *ER* endoplasmic reticulum. Created with Biorender.com

Substantial laboratory findings have revealed and supported an intimate biological connection between the liver and eyes under physiological and pathological conditions, despite their great anatomical distance. We summarized consistent molecular regulatory mechanisms and cross-talking pathways between the two organs (Figs. 4, 5).

### Consistent biological regulatory patterns of the liver and eyes

Normal retinal pigment epithelium (RPE) consists of a monolayer of melanin-rich epithelial cells located between choroids and photoreceptor (PR) outer segments, which works with PR to sustain visual cycles [57]. Epithelial-mesenchymal transformation (EMT) transforms RPE cells to de-differentiated mesenchymal phenotypes (loss of apical-basal cell polarity, dysregulated cell proliferation and migration, and blindness correlation) [58]. In vitro, RPE cells originated from human induced pluripotent stem cells (hiPSC-RPE) show the enrichment of liver tumor/carcinoma/carcer tox functions-related proteins and phosphosites alterations as well as sharing commonality with hepatocyte proliferation-initial stages of liver malignancy-related pathways [57–59]. Overall, the highest enrichment of hepatocyte growth factor-cellular-mesenchymal to epithelial transition factor (HGF-MET) signaling pathway has been detected, which could regulate EMT transcriptional profiles [57].

Besides, the retina is as metabolically active as the liver parenchyma in the protein synthesis and such an anabolic pattern can be attributed to constant insulin receptor and Akt-1 kinase activities comparable to those of the postprandial liver [60]. In addition, RPE cells have been found to express as high levels of ketogenesis and fatty acid oxidation-related mitochondrial HMG-CoA synthase 2 (Hmgcs2) to generate β-hydroxybutyrate through fatty acid oxidation for energy supply as hepatocytes do [57, 61, 62].

### Glucose metabolism

Liver plays a central role in blood glucose homeostasis, as it involves in regulating multiple pathways of glucose metabolism, including glycogenolysis, gluconeogenesis, glycogenesis, and glycolysis [63]. Besides, the liver could regulate insulin efficacy through removing 50% of secreted insulin in peripheral circulation, inhibiting hyperinsulinemia induced insulin resistance in adipose/muscle tissues [64, 65]. Liver glucose supply matches whole-body glucose demand (80–90% of endogenous glucose production derived from the liver under postabsorptive condition) [66], and a relatively stable level of blood glucose is the major energy source for normal retina metabolism [67].

In acute or chronic liver disease, hepatocytes could not respond to insulin signaling in a physiological way and resultant dysregulated glycogenolysis, gluconeogenesis, and lipogenesis promote hyperglycemia, systemic insulin resistance and eventually causing elevated risk of T2DM, of which NAFLD is the most prevalent type and lead to a nearly two-fold increased risk [68, 69]. Lipid intermediates accumulation in liver impairs its ability of insulin clearance and induces hepatic insulin resistance as well as gluconeogenesis [69].

Blood glucose in the hyperglycemic condition enters the polyol pathway and is reduced to sorbitol by aldose reductase in the eye lens, causing apoptosis and eventually cataract [70, 71]. Besides, hyperglycemia can result in abnormal metabolism in vascular endothelial cells of the eyes to impair them, and an activated pro-inflammatory phenotype of retinal microglia, Müller cells, and migrated circulating leukocytes in retinal microcirculation,

which collectively cause vascular bed dysfunction and chronic regional inflammation [72, 73]. Recently, it has also been found that high levels of blood glucose could cause injuries directly on neuronal cells (neuroretina), even prior to the breakdown of the blood-retinal barrier (BRB), to promote neurodegeneration, neuronal cell death, and eventually DR [74].

Furthermore, insulin resistance and hyperglycemia stimulate sympathetic nerve activity as well as trabecular meshwork cell excessive synthesis of extracellular matrix to cause increased IOP [75, 76]. Apart from hyperglycemia-related toxicity, some secreted molecules might also play a role in communications between the liver and the eyes. In infants with ROP, increased incidence and severity of this illness may be due to subdued endogenous insulin signaling-induced liver insulin-like growth factor 1 (IGF1) reduction and use of IGF1 exerts significant inhibition on pathological neovascularization and improvement of physiologic retinal revascularization [77]. Likewise, fibroblast growth factor-21 (FGF-21) is another regulatory factor primarily excreted by hepatocytes, of which the level is decreased in T2DM patients [78]. Supplementation of FGF-21 has shown inhibitive effects on retinal neovascularization in mice mimicking hypoxia-caused neovascularization in DR [79–81].

### Amino acid and protein metabolism

Liver metabolized amino acids and proteins impose impacts on the eyes under both physiologic and pathologic scenarios. Taurine is a sulfur-containing amino acid residing and functioning throughout the retinal layers particularly in the RPE and PR cells [82], and its deficiency leads to PR degeneration and retinal ganglion cell loss [83]. It has been found that taurine in the retina is primarily synthesized from liver glycolysis-derived 1-carbon than from that of retinal glycolysis, under the control of liver HIF-1α stabilization [84, 85].

Ornithine aminotransferase (OAT) is an enzyme predominantly expressed by liver cells, and involves in the catabolism of ornithine to proline precursors [86]. Its deficiency causes hyperornithinemia at 10–20 folds of the normal levels, and the cytotoxicity of excessive free ornithine would cause gyrate atrophy of the choroid and retina [87].

Besides, newly synthesized retinol-binding protein 4 (RBP4) could bind with and then transfer retinol in serum, while the deficiency causes immobilized liver storage, reduced serum levels of retinol, and a disrupted visual cycle [88, 89], its excess could also impair retina via microglia and IL-18-mediated inflammation in mice models [90, 91]. RBPR2 is a liver-specific receptor for the RBP4-retinol complex, which mediates the liver and systemic circulation retinol cycle [92]. The RBPR2^−/−^ mice model exhibits reduced liver storage and ocular supplies of retinoids, as well as a significant loss of visual ability [92]. Liver Kupffer cells are the main source of HGF, and this cytokine could bind with its receptors to sustain the structural and functional integrity of corneal/lens epithelial cells, ganglion cells, and RPE cells [93, 94], as well as hyperactivate the MET receptors overexpressed by uveal melanoma (UM) cells to facilitate metastasis and therapy resistance [95]. Furthermore, ABCC6 is an organic substrate transporter expressed exclusively by hepatocytes, while its pathogenic mutation might alter liver secretion of anti-mineralization/anti-calcification proteins, like fetuin-A and Gla proteins, and cause eye mineralization in pseudoxanthoma elasticum (PXE) [96]. Reduced albumin generation by the liver results in low intravenous oncotic pressure that induces retinal soft exudates extravasation [97].

In turn, retinal pigment epithelium-derived factors (PEDF) excreted by RPE could act on the liver [98]. It has shown systemic impacts on inhibiting the Wnt coreceptors, low-density lipoprotein receptor-related protein 6 (LRP6) and steatohepatitis severity [99, 100].

### Fatty acid, cholesterol, and bile acid metabolism

The liver participates in systemic metabolism and circulation of lipids to modulate ocular pathophysiological bioactivities. As long ago as in 1980s, the liver was found to convert 18:3 omega-3 fatty acid to 22:6 omega-3 fatty acid, and then this docosahexaenoic acid was transported in secreted lipoproteins to the developing retina to synthesize membrane phospholipids [101, 102]. Also, the liver cytochrome P450 epoxygenase-derived epoxyeicosatrienoic acid (EET) exerts an inhibitive effect on eye inflammation [103]. As to cholesterol, RPE contains an HDL-based active reverse transport system that could return excessive peripheral cholesterol to the liver [104]. The primary bile acids (BAs) are liver metabolites of cholesterol, which contribute to the absorption of vitamin A and dietary fat, and they undergo deconjugation and dehydroxylation to secondary BAs (i.e., deoxycholic acid [DCA] and lithocholic acid [LCA]) in the distal part of small intestine and colon [105]. This conversion is partially mediated by the modification of gut bacteria [106].

In NAFLD patients, however, the abundance of the bacteria responsible for conversion is decreased, which leads to decreased stimulation of BAs receptors by secondary BAs and further intestinal microbial disturbance [107]. Reshaping intestinal microbiome is accompanied by altered levels of secondary BAs [108]. The ursodeoxycholic acid (UDCA) and tauroursodeoxycholic acid (TUDCA) of the secondary BAs exert neuroprotective impacts on retinopathies mainly via activating the TGR5-mediated pathway [109].

### Provitamins, vitamins, and their derivatives metabolism

Carotenoids refer to a group of natural, orange/yellow/red color, lipophilic, natural pigments, in which the Provitamin A subtype (β-carotene, α-carotene), and retinyl esters (RE) are absorbed and transported to the liver, where they are partially stored in liver stellate cells as RE (retinyl palmitate and retinyl stearate) or converted to all-trans retinol which binds with RBP4 to be secreted into the bloodstream [110]. Meanwhile, the rest of the unprocessed carotenoids (lutein, zeaxanthin, and β-carotene) are integrated into LDL and HDL to be returned into the circulation [111]. In RPE cells, retinol is transformed to 11-cis retinol, the precursor of 11-cis retinaldehyde by RPE65, which constitutes rhodopsin with opsin to retain the photosensitivity of rod cells [111]. Meanwhile, circulated zeaxanthin and lutein are utilized by the retinal macula to filter harmful blue light as well as to repress singlet oxygen species-caused oxidative stress [112], in which HDL (specifically ApoA-I) answers for such courier [113]. Furthermore, vitamin A is transformed to its active derivative retinoic acid on ocular surface, which activates the retinoic acid receptor (RAR) and retinoid X receptor (RXR) to ensure normal differentiation and mucus secretion of corneal and conjunctival epithelial cells [114, 115]. Interestingly, misfolded rhodopsin can cause retinal oxidative stress that in turn undermines hepatic antioxidative defense in P23H transgenic rats mimicking retinitis pigmentosa (RP), potentially through damaging the melanopsin system that causes circadian desynchronization or certain retina-derived, liver-directed molecules [116].

Besides, the liver is an important organ for synthesizing B vitamins, including folic acid [117], which could protect retinal ganglion cells from death in glaucoma and prevent retinal microvascular endothelial cell from DNA methylation/hydroxy-methylation impairment in DR [118–120].

In addition, vitamin D activation undergoes two rounds of hydroxylation, of which the first step of hydroxylation to 25(OH)D is catalyzed by CYP2R1 in the liver [121, 122]. Patients with hepatic diseases are largely accompanied by reduced liver CYP2R1 activities and thus lower blood vitamin D levels [1, 123], so does those with genetical variants within the CYP2R1 locus [40].

### Mineral metabolism

The systemic homeostasis of human-required minerals, like iron, copper, and zinc, highly depends on the normal liver function, while liver disease can impair such balance and impose great impacts on eye functions [124]. Iron is abundant in the retina and indispensable for essential biochemical activities, while an excessive intracellular load of it could cause oxidative stress and ferroptosis [125]. Hepcidin (Hepc) is the key hormone for reducing the blood concentration of iron, which is primarily synthesized by the liver and functions through antagonizing the only known. iron exporter ferroportin (Fpn) in human to inhibit intestinal absorption and liver release of the iron [126]. Although the retina has a regional regulatory mechanism by synthesizing Hepc just like hepatocytes do, transgenic mice model indicates that liver-specific rather than retina-specific absence of Hepc leads to increased blood and retinal free iron levels that enable subsequent hypertrophy of RPE and degeneration of PR cells [127]. Zinc is the second most prevalent essential trace element *in vivo* [128], with its content being particularly high in ocular tissues (with the highest level in the retina-choroid) [129]. Zinc is indispensable for many essential physiological processes of the retina, including regulating rhodopsin stabilization and retinol metabolism, while its systemic deficiency may lead to retinitis pigmentosa as well as abnormal visual dark adaptation [129, 130]. It has been reported that alcoholic/viral liver disease could result in zinc deficiency in patients along with corresponding ocular manifestations [128].

Hepatolenticular degeneration is an inborn defect of copper metabolism caused by pathological mutation of the transmembrane copper-transporter ATPase gene ATP7B [131]. The generated dysfunctional ATP7B copper transporters could result in impaired biliary copper excretion, and the resultant excessive deposition of copper in ocular tissues leads to corneal pigment ring (the Kayser-Fleischer ring), nystagmus, or sunflower cataract [132].

### Detoxification of ammonia and bilirubin

Cell metabolism bioactivities in vivo, like amino acid deamination/transamination, purine/pyrimidine decomposition, and urea degradation by gut microbiota, can produce nearly 1000 mmol of ammonia per day, of which the majority is converted by the liver. to urea and then removed in the form of urine to sustain a normal plasma level (in adult, < 50 μm; in neonate, < 150 μm) [133]. Besides, liver converting glutamate into glutamine by glutamine synthetase and amino may well represent another equally important pathway of removing ammonia [134]. Liver disease could disrupt these processes and then resultant. high plasma ammonia (0.2-1 mM) could obstacle the citric acid cycle and mitochondrial respiratory chain, causing great toxicities to retinal Müller cells (vacuolization, swelling, and even necrosis) as well as to optic nerves [135–137]. The nitrogen metabolism patterns of retina that primarily rely on glutamine synthesis to remove ammonia make it vulnerable to ammonia toxicity [133].

Similarly, liver disease undermines the clearance ability of hepatocytes of unbound bilirubin from the blood and the resultant high plasma levels of such a potent neurotoxin could bind with and yellow the conjunctiva (one of the most visible manifestations) and cause retinopathy [138], potentially by affecting transporters of the blood-retina barrier [139–141]. In addition to the cytotoxicity to eye tissues, hyperbilirubinemia could cause injuries to visual cortex and impair visual acuity (called hepatic cortical blindness) [142]. However, the negative effect of unbound bilirubin on eye health is not always definite, as its potent antioxidative property may exert protective effects upon specific conditions mentioned before [42, 143].

### Immunity regulation

Liver is the major production pool of complements [144], and its synthesized complement factor H (CFH) prevents the alternative pathway of complement activation, and membrane attack complex formation/deposition in choroidal neovascularization [145].

**Fig. 6:**
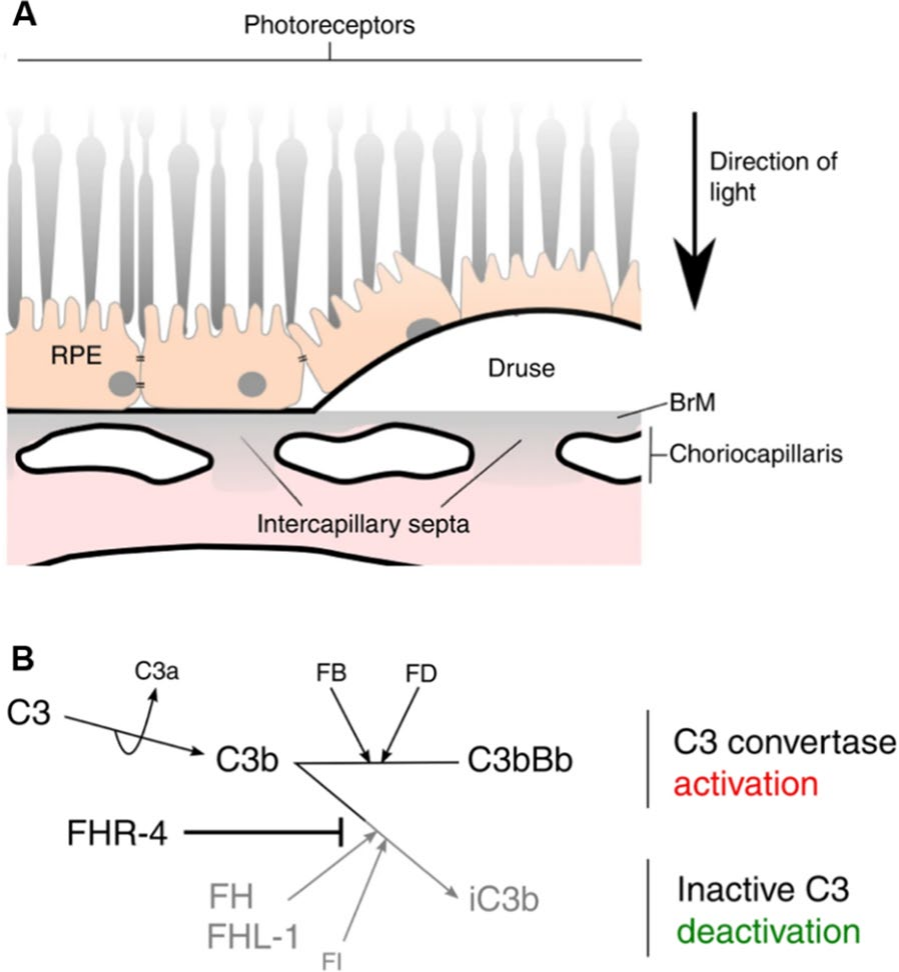
FHR-4 accumulated in the choriocapillaris could inhibit C3b breakdown. **A** The diagram illustrates the anatomical structures of the macula, including the RPE, the underlying BrM, and the intercapillary septa within the choriocapillaris. Basement membranes are shown in black lines. Drusen is the hallmark lesion of early AMD that forms in the BrM underneath the RPE basement membrane. *RPE* retinal pigment epithelium, *BrM* Bruch’s membrane, *AMD* age-related macular degeneration. **B** FHR-4 prevents FHL-1 from acting as a cofactor for factor I and results in C3 convertase formation and subsequent activation of the amplification loop of complement and inflammation. FHR-4, Factor H related protein 4, FHL-1, FH-like 1. **A** and **B** are adapted from Fig. 2a and Fig. 2j in [16], Cipriani, V., Lorés-Motta, L., He, F. et al., Increased circulating levels of Factor H-Related Protein 4 are strongly associated with age-related macular degeneration. Nat Commun 11, 778 (2020), with authorization from the Creative Commons International licence 4.0. (http://creativecommons.org/licenses/by/4.0/)

Besides, the RNA sequencing has indicated that in vivo the only source of FHR-4 is the liver and that FHR-4 accumulation in the choriocapillaris can stimulate the complement system and recruit circulating immune cells to exacerbate inflammation [146] (Fig. 6).

Besides, liver dysfunction leads to increased intestinal permeability, gut bacterial translocation, as well as serum LPS and various pathogen-associated pattern molecules (PAMPs) levels, which promotes the secretion of pro-inflammatory cytokines TNF-α and IL-6 by adipose tissues [147]. Such elevated release of pathogenic mediators has been linked with retinopathy by activating microglia infiltration through pattern recognition receptors (PRRs) [30, 148]. Likewise, fetuin-A is a glycoprotein predominantly synthesized and secreted from the liver that enhances the secretion of proinflammatory cytokines in adipose tissue as well, while NAFLD patients have elevated serum levels of it [149].

### Other modulatory pathways

Melanocyte-derived exosomes obtained from hepatic circulation of UM patients with liver metastases contained microRNAs (miRNAs), of which the upregulated miR-454, let-7b, and miR-21 are involved in regulating liver stellate cell activation [150–152].

Excessive estrogen due to liver dysfunction might protect the eyes by alleviating PR cell apoptosis in RP mice through the classic estrogen receptors (ERs)-mediated N-myc Downstream Regulated Gene 2 expression routine [153], by protecting retinal astrocytes along with ganglion cells from endoplasmic reticulum stress via activating G-protein-coupled ER in ROP [154, 155], by inhibiting Caspase-3 activation and tau protein dephosphorylation to protect astrocytes from oxidative stress [156], and by modulating the mitochondrial pathway to reduce high-glucose caused retinal ganglion cell damages [157].

In addition, the gut (including intestinal microbiota and their products) could be mediators of liver-eye communication, as liver diseases regulate their homeostasis, which is intimately correlated with ocular pathogenesis [158]. It has been found that valeric acid, a gut microbiota metabolite, could penetrate to the eyes and sustain homeostasis of IOP, while NAFLD patients were less abundant in valerate [159, 160]. Besides, decreased butyrate production is found in NAFLD [161], while butyrate has shown protective effects on ocular surface inflammation [162], DR [163], and intraocular bacterial infection [164]. Moreover, activating the aryl hydrocarbon receptor (AHR) and its involved signals is associated with the protection of RPE cells and the retina, as well as the inhibition of choroidal neovascularization, uveitis, and AMD [165]. NAFLD-related dysbiosis has been proven to cause decreased indole and its derivatives production, while these factors are endogenous ligands of AHR [165, 166].

## Clinical links and practical applications

### Diagnostic techniques

Visible alterations in eye manifestations can help clinicians to timely diagnose and deal with liver-related health issues, like Kayser-Fleischer ring implying Wilson disease, sclera icterus reflecting jaundice, xanthelasma palpebra indicating hepatic steatosis, and spontaneous subconjunctival/vitreous/retinal hemorrhage referring to hepatic failure [1, 2].

Besides, clinical statistics could provide clues for relatively hidden illnesses, for nearly 50% of cases of gram-negative endogenous endophthalmitis (EBE) originate from liver abscesses [167], and patients with orthotopic liver transplants are susceptible to Aspergillus endophthalmitis with eyes being the second most common site of infection only to lung [168]. In addition, nearly half of UM patients develop metastases, with the liver being the most preferential site [95, 169]. Nevertheless, the non-specificity and non-sensitivity of such summarized clinical features undermine their value as independent diagnosis index [2, 170].

**Fig. 7:**
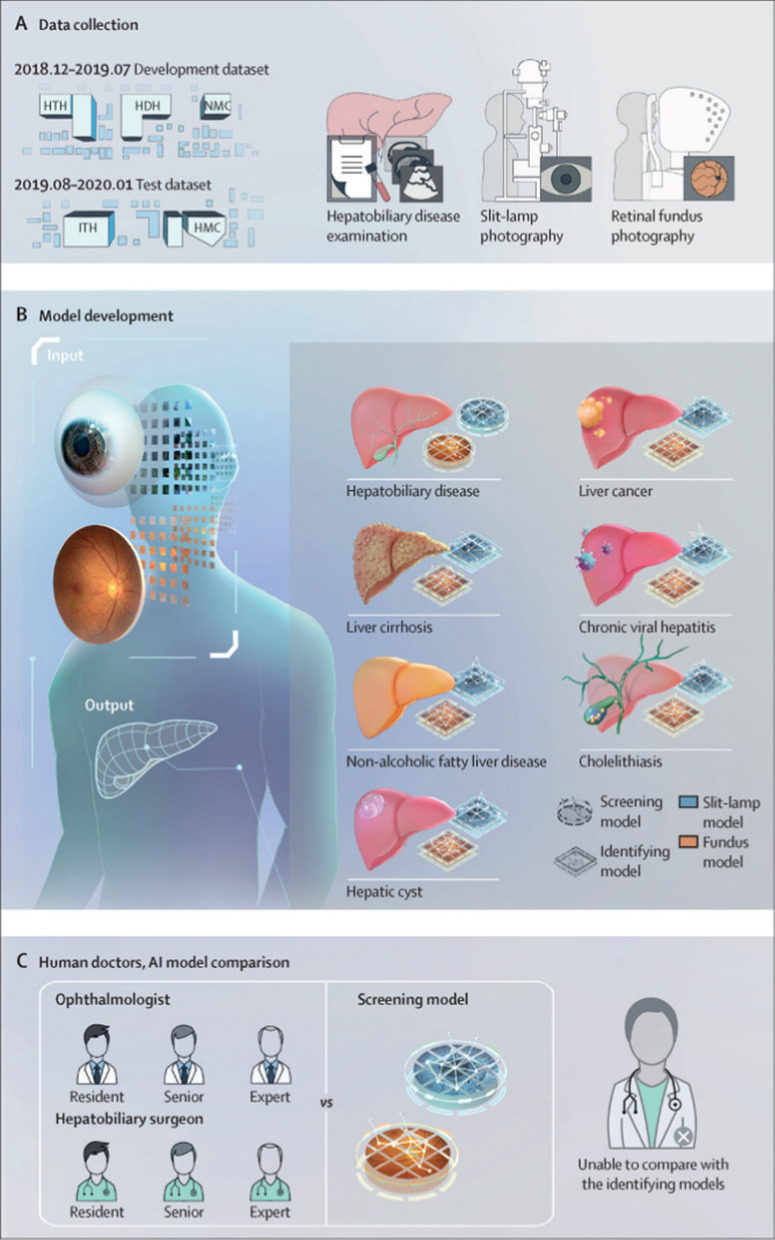
The schemes for constructing the AI-DL-based liver disease diagnosis models. **A** Collection of liver disease examination results, slit-lamp, and retinal fundus images. **B** Slit-lamp images and retinal fundus images are categorized to train DL algorithm-based models for identifying liver disease separately **C** Competitions between human clinicians and AI-DL models, involving three ophthalmologists, and three hepatobiliary surgeons. *AI* artificial intelligence, *DL* deep learning. Reprinted from [2], Xiao W, Huang X, Wang JH, et al. Screening and identifying hepatobiliary diseases through deep learning using ocular images: a prospective, multicentre study. Lancet Digit Health. 2021; 3(2): e88–e97 without any adaption, Copyright (2021), with permission from Elsevier under the Creative Commons (CC-BY-NC-ND 4.0) license (https://creativecommons.org/licenses/by-nc-nd/4.0/)

In recent years, the video electro-oculography (VOG) technique designed for early detection of dysfunctional cognitive/motor abilities in Parkinson’s, Alzheimer’s disease or multiple sclerosis has also been tested in diagnosing minimal hepatic encephalopathy (MHE), the earliest form of hepatic encephalopathy commonly found in liver cirrhosis patients [35, 171–173]. The results showed that MHE patients have longer latencies and worse performance in most eye movement tests, of which the latency of reflexive saccades in vertical antisaccades test and the vertical version of the memory-guided saccades test [35]. The underlying mechanism relates to impaired mental processing speed and attention [35]. In addition, artificial intelligence (AI)-based deep-learning (DL) algorithm has evolved rapidly in medical imaging-processing by automatically analyzing input graphs and coming to diagnosis data [174, 175]. This technology has been validated in image-centered ophthalmology to detect glaucoma, multiple retinopathies (including ROP, AMD, DR, and diabetic macular edema) [176–180]. The latest study adopts the ResNet-101 deep neural network to establish both slit-lamp and fundus images-trained DL models, in which the slit-lamp model performs well in detecting liver cirrhosis and cancer, while both models work relatively weaker in predicting cholelithiasis, NAFLD, viral hepatitis, and hepatic cysts [2]. Interpretation of working principles indicates that the structure of the iris, conjunctiva, sclera, and fundus contains diagnostic information identifiable to AI-DL models (Fig. 7) [2].

### Therapeutic strategies

**Fig. 8:**
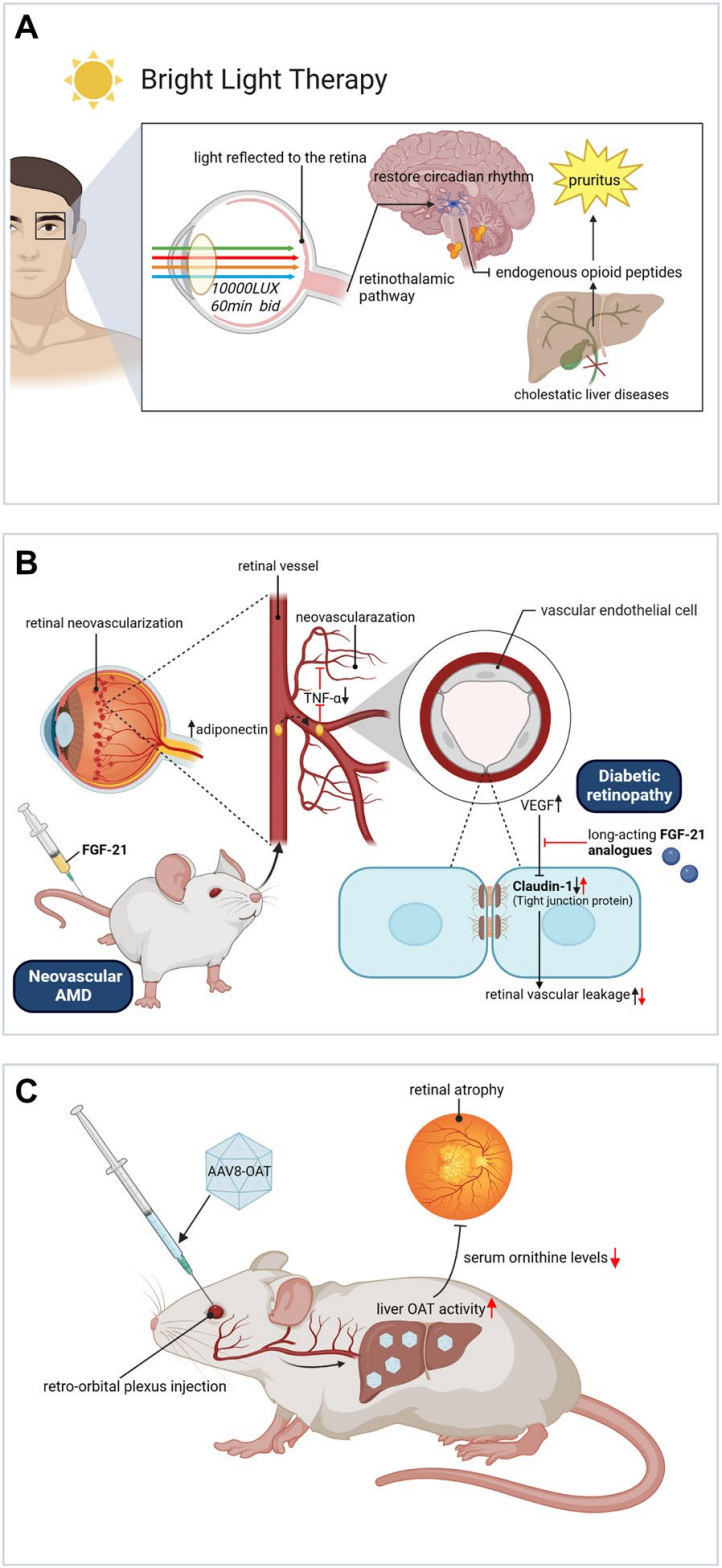
Intimately correlated therapeutic pathways. **A** The schemes of using eye-directed bright-light therapy against pruritus via restoring circadian arrhythmia-related endogenous opioid dysregulation. The light density is 10,000 LUX, with the frequency at 60 min each time, bid. **B** Long-acting FGF-21 could counteract increased VEGF-induced retinal vascular leakages in DR via increasing inter-endothelial tight junction protein Claudin-1 expression levels. FGF-21 can inhibit retinal neovascularization and inflammation via an adiponectin-dependent way in AMD murine models. *FGF-21* fibroblast growth factor-21, *VEGF* vascular endothelial growth factor, *AMD* age-related macular degeneration, *DR* diabetic retinopathy. **C** OAT-carrying AAV8 vectors are administered through intravenous retro-orbital plexus injections to restore liver OAT levels. Restored liver OAT activity can reduce serum ornithine levels to treat choroidal and retinal gyrate atrophy. *AAV8* adeno-associated virus serotype 8, *OAT* ornithine aminotransferase. All pictures are created with BioRender.com

In addition to diagnosis, hepatic and eye diseases also exhibit intimate associations in therapy, indicating that targeting at the liver could help treat ocular abnormalities and vice versa (Fig. 8).

Pruritus is an agonizing complaint frequently from patients with cholestatic liver diseases, such as primary biliary cirrhosis and primary sclerosing cholangitis. A clinical trial has validated the amelioration effects of bright-light therapy (BLT) on hepatogenic pruritus, potentially via restoring circadian rhythms through retino-thalamic pathway [181, 182]. For liver abscess-induced EBE treatment, early surgical resection of primary lesions and administration of antibiotics are essential for recurrent patients [183]. In addition, liver transplantation could restore disrupted electroretinogram and blue-yellow defects in the eyesight of patients with grievous liver failure [115]. The administration of FGF-21 could prevent retinal or choroidal neovascularization and regional TNF-α expression through upregulating adiponectin in circulation as well as retina in neovascular AMD models [80].

Also, the use of IGF1 holds potential for preventing ROP, as previously referred [77]. In recent years, emerging gene therapy has given hope of potential cure for various diseases, of which the eye is a prime target [184]. In current studies, adeno-associated viral vectors (AAVs) have been the priority vectors of choice and their common hepatotropic properties enable effective gene transfer towards hepatocytes to restore their generation of deficient proteins [184, 185]. For OAT (EC 2.6.1.13) deficiency, current therapy strategies of reducing plasma ornithine levels by arginine-restricted and vitamin B6-enriched diet merely slow but not prevent gyrate atrophy of the choroid and retina [86]. Using serotype 8 AAV (AAV8) vector, a preclinical work suggested that restoration of a minimum 10% of liver specific OAT activity could reach a persistent decrease in serum ornithine levels as well as a significant inhibition of retinal degeneration [87, 186]. Likewise, using antisense targeting at liver CFHR4 synthesis might become promising for AMD treatment [187]. In addition to the gene transfer technology, herbal medicinal components show therapeutic potentials as well [188]. The Traditional Chinese Medicine (TCM) adopts principles: “clear liver heat to enhance eyesight”, “liver blood deficiency inducing myopia”, and “nourish liver-yin to improve visual function” to guide eye disease therapy, and TCM physicians therefore prescribe black or brown bear bile (containing the major effective component TUDCA), medicinal herbs like wolfberry (containing lycium barbarum polysaccharide), aloe vera (containing aloin), acupuncture, or compound preparations (Qiming granules) to ease eye discomforts complicated with “liver depression” [135, 189–192]. Nevertheless, a prospective study of T2DM patients using glucagon-like receptor-I agonists calls for more attention paid on potential side effects of therapeutic strategies, as a protective effect on liver steatosis of NASH was found with aggravated retinopathy simultaneously [71]. Current and future treatments for liver diseases should guarantee that they would not worsen ophthalmopathy (if existed) and *vice versa* [193].

## Conclusions

This review tries to preliminarily link two anatomically and functionally irrelevant organs together individually from the epidemiological, mechanical, and clinical aspects.

Particularly, it is worth noting that various pathways might cooperate with or counteract each other, like hepatic saturation of free fatty acid could elevate iron stores, which then amplifies T2DM-related retinal pericyte loss [64]. Also, the COVID-19 pandemic-caused mandatory lockdown further stressed the necessity of combining AI-DL-based models with 5G-based Cloudy medicine to remotely screen for disease in a noninvasive as well as convenient manner [2, 194].

Regarding liver-eye interactions, there are still certain unsolved problems worth further exploration. In terms of the epidemiological aspect, current work tends to simply show changes in disease severity or prevalence rates rather than calculate the precise thresholds of liver disease indices alterations enabling to predict the occurrence or stages of ophthalmopathy or those of ocular indices enabling to judge hepatic status. Therefore, further research may well adopt larger patient-cohorts to confirm potential correlations and provide references for public health policymaking. As to the molecular mechanisms field, functions of liver/eyes-specific molecules on targets, along with their space–time regulatory/transporting mechanisms under both pathological/physiological situations, remain to be clarified, particularly for those with regional regulatory systems in the eyes or those with ectopic expression, such as leucine-rich α-2 glycoprotein 1 (LRG-1), a constitutive liver protein that is also strongly expressed by eyes in pathological situations [195]. Besides, feedback signals from target organs and their reciprocation with primary organs/signals are still unknown, like how the eye signals to the liver to modulate its vitamin A store release when concentrations of retinoid are low. In clinics, TCM theories and therapies-entailed scientific foundation could help researchers focus on the liver-eye axis from a holistic view and should receive more attention, but the liver toxicity of ethnomedicine requires extra attention [196]. Meanwhile, current AI-DL models are still suboptimal in milder liver disease detection and clinical utility, but their relatively-high sensitivity in identifying early-stage liver pathologies still holds promise for becoming a diagnostic tool. All in all, more studies focusing on liver-eye interorgan communications would enhance our understanding of their underlying molecular regulatory pathways and help us to develop rational early detective/therapeutic methods to reduce disease burden and improve clinical prognosis.

## Data Availability

All data/graphs adopted are available from corresponding authors upon proper request.
